# Limitations of the Spike-Triggered Averaging for Estimating Motor Unit Twitch Force: A Theoretical Analysis

**DOI:** 10.1371/journal.pone.0092390

**Published:** 2014-03-25

**Authors:** Francesco Negro, Utku Ş. Yavuz, Dario Farina

**Affiliations:** 1 Department of Neurorehabilitation Engineering, Bernstein Focus Neurotechnology Göttingen, Bernstein Center for Computational Neuroscience, Georg-August University of Göttingen, Göttingen, Germany; 2 Department of Orthobionics, Bernstein Focus Neurotechnology Göttingen, Bernstein Center for Computational Neuroscience, Georg-August University of Göttingen, Göttingen, Germany; The University of Queensland, Australia

## Abstract

Contractile properties of human motor units provide information on the force capacity and fatigability of muscles. The spike-triggered averaging technique (STA) is a conventional method used to estimate the twitch waveform of single motor units *in vivo* by averaging the joint force signal. Several limitations of this technique have been previously discussed in an empirical way, using simulated and experimental data. In this study, we provide a theoretical analysis of this technique in the frequency domain and describe its intrinsic limitations. By analyzing the analytical expression of STA, first we show that a certain degree of correlation between the motor unit activities prevents an accurate estimation of the twitch force, even from relatively long recordings. Second, we show that the quality of the twitch estimates by STA is highly related to the relative variability of the inter-spike intervals of motor unit action potentials. Interestingly, if this variability is extremely high, correct estimates could be obtained even for high discharge rates. However, for physiological inter-spike interval variability and discharge rate, the technique performs with relatively low estimation accuracy and high estimation variance. Finally, we show that the selection of the triggers that are most distant from the previous and next, which is often suggested, is not an effective way for improving STA estimates and in some cases can even be detrimental. These results show the intrinsic limitations of the STA technique and provide a theoretical framework for the design of new methods for the measurement of motor unit force twitch.

## Introduction

The contractile properties of a muscle depend on the mechanical characteristics of its functional elements, the motor units [1]. During a contraction, the discharges of the motor neurons generate trains of twitch forces of the innervated muscle fibers, whose summation results in the total force produced by the muscle [2]. The identification of the individual twitch temporal profiles is important for the study of the contractile muscle properties [3], [4]. The spike-triggered averaging (STA) is currently the only technique used to estimate the properties of the motor unit twitch forces *in vivo* during voluntary contractions [5], [6], [7], [8], [9], [10], [11]. This technique consists in averaging the joint force signal, using the discharge times of a motor unit as triggers. The main assumptions of the method are the linear summation of twitch forces, the uncorrelated activity of concurrently active motor neurons, and the duration of the twitch smaller than the inter-spike interval. Under these assumptions, the averaging increases the signal-to-noise ratio (SNR) for the force expressed by the trigger unit with respect to the other active motor units. However, the above assumptions are not fully met in practice and their violation has been proven to bias the estimates substantially [12], [13], [14], [15]. For example, if the time duration of the twitch force is longer than the average inter-spike interval of the trigger unit, the estimate is biased by fusion of the twitches.

Although the characteristics of the STA have been investigated extensively in simulation [13], [16], [17] and experimental studies [6], [18], there has not been a general theoretical analysis that underlined the intrinsic limitations of this technique. A deeper analytical analysis of the method would lead not only to the identification of its theoretical limits, but also to the possibility of developing improved techniques. Some attempts in this direction have been performed [19], [20], but they were focusing on some particular applications of the technique. Therefore, in this study, we provide a more general theoretical study of the STA technique applied to the estimation of single motor unit twitches, together with a description of its limitations. In particular, we show that the correct estimation of the force twitch of a single motor unit during voluntary contractions in physiological conditions is theoretically bounded by several factors that cannot be overcome. New approaches to improve the ability for accurately assessing contractile properties at the single motor unit level should consider these limitations and compensate for the factors of influence [17], [19], [21].

## Materials and Methods

### Theory

The generation of the force signal can be modelled as a spatial and temporal summation of motor unit twitch forces:
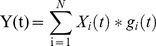
(1)where *X_i_(t)* and *g_i_(t)* are respectively the train of motor neuron discharge times and the twitch force of the *i-th* motor unit. The stochastic processes are here indicated with capital letters. *N* is the total number of active motor units. In this theoretical model, we assume that *g_i_(t)* does not depend on the time of the activation of the preceding twitches, although this assumption does not hold in general [2], [22], [23], [24]. Inclusion of time-varying twitches would include additional term of variability in the estimate without invalidating the main conclusions [20]. In order to estimate *g_i_(t)*, the STA method requires the knowledge of the activation pattern *x_k_(t)* of the *k*-th motor unit. In this case, we obtain:
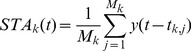
(2)where tk,j is the instant of the j-th discharge of the k-th motor unit and Mk the total number of its discharges. Disregarding the normalization coefficient 1/Mk and assuming that the number of triggers Mk tends to infinity (spike train of infinite duration), Eq. 2 is equivalent to the cross-correlation between the k-th spike train and the force signal [25] and therefore can be re-written as:



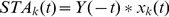
(3)The stochastic estimate *STA_k_* is the convolution of the random process *Y(t)* (force) by the deterministic (because it is obtained by a measurement and is assumed to be known) spike train of the *k*-th motor unit. The power spectrum of *STA_k_* is the product of the power spectrum of the force and the energy spectrum of the spike train:
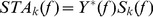
(4)where *Y(f)* is the power spectrum of the force signal and *S_k_(f)* the energy spectrum of the spike train used in the STA calculation.

Including Eq. 1, Eq. 4 becomes
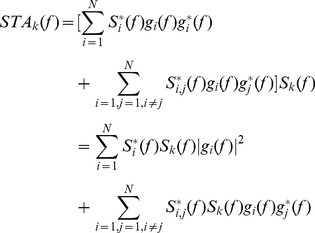
(5)


Eq. 5 states that the STA estimation of the *k*-th motor unit twitch in the frequency domain is given by one term that contains the spectrum of the *k*-th spike train multiplied by the sum of all auto-spectra of the motor unit twitch trains that constitutes the force signal. The second term contains the information related to the cross spectra of the motor unit twitch trains. This expression can be rewritten as
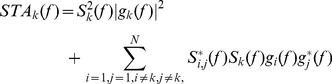
(6)


Eq. 6 indicates that a reliable estimate depends on the relative power of two terms: the first relates to the information of the triggered motor unit twitch and the second derives from the activity of the remaining *N-1* motor units. Under the assumption of uncorrelated trains, we obtain:
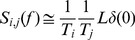
(7)where *T_i_* and *T_j_* are the mean interspike intervals of the two spike trains and *L* the total duration of the signal. Therefore, when the spike trains are uncorrelated, the second term is simply an additional noise term (auto spectra part) summed to a DC value (cross spectra part) that decreases the SNR of the estimation. However, a low to moderate level of synchronization is usually found during voluntary contractions [26], [27]. In these conditions, this second term depends on the duration of the signal, the number of motor units *N*, and the correlation level, as analysed in the Results.

In the following derivations, we will indicate the second term due to correlated discharge trains as *C(f)*. In the following derivations we will assume that its contribution is relatively small (completely uncorrelated spike trains and very high numbers of triggers) and it can be neglected. With this notation, we can write Eq. 6 as:
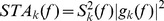
(8)


In this last expression, the twitch transfer function is multiplied by the square of the spectrum of the spike train used as a trigger. This implies that the spectral characteristics of the spike train belonging to the motor unit investigated influence the estimation.

In the simplified assumption that the target motor unit spike train is exactly periodic, we can write
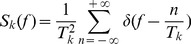
(9)where *T_k_* is the period of discharges.

In this case, Eq. 8 is equivalent to the sampling in the frequency domain of the twitch waveforms, generating a sum of line spectra:
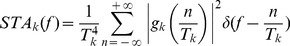
(10)


In this case, the information of the twitches is all contained in the discrete line spectra that are confined in the bandwidth of the twitch waveforms. The greater the number of delta functions within the bandwidth of the twitch, the better the twitch representation (which is equivalent to state that the interspike interval should be long enough to contain the twitch duration). Therefore, the pulse frequencies *1/T_k_* and the frequency support of the twitch waveforms *g_k_(t)* dictate the representation of the original twitch waveforms in the output force signals and hence the possibility of estimating the former from the total force output. Since the bandwidth of the motor unit twitches is relatively small, this phenomenon produces an important limit for the correct estimation of its parameters in case of periodic spike trains. Only the use of very long interspike intervals would provide an accurate estimate.

During voluntary activation of a muscle, the motor unit pulse trains are not periodic but can be modelled as a quasi-periodic spike train with moderate level of inter-spike interval (ISI) variability (coefficient of variation, CoV, for ISI approximately 15%). In this case, the spectrum of the k-th pulse train can be written as [28], [29], [30]:
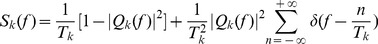
(11)where *Q_k_(f)* is the Fourier transform of the probability density function of the ISI variability and *T_k_* the average period of the ISI. Eq. 11 shows that a quasi-periodic pulse train has two frequency components: one continuous spectrum that depends on the Fourier transform of the probability density function *Q_k_(f)* of the ISI, and a line spectrum similar to the periodic case, but with amplitudes weighted by the Fourier transform of the statistic distribution of ISI. For simplicity, we focus on the square root of the STA estimation. From Equation 8 we obtain:



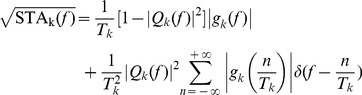
(12)The continuous term in the power spectrum of the STA improves the sampling of the twitch transfer function, with respect to the pure periodic case, because it has a large bandwidth and provides a proportional term for all frequencies. In this case, the sampling limit described for the periodic spike train does not apply and the accuracy of the sampling depends on the relative amplitude of the continuous spectrum. For this reason, with an appropriate degree of variability, the STA estimate can be accurate even with high discharge rates. Appendix S1 reports the derivation of the optimal coefficient of variation for ISI when the twitch force is modelled as proposed by [2] and the discharge times are normally distributed.

### Simulations

Simulations were performed using the twitch waveform described by the equation proposed in [2]. In most simulations, the peak amplitude (P) was fixed to 1 arbitrary unit (AU), whereas the time to peak (T) was chosen between 30 and 90 ms. In one simulation, the motor units were simulated with an exponential variation of P and T [2]. The rising time of the smallest motor unit was set to 90 ms. The range of twitch tensions was set to 100 and the range of contraction times was set to 3 [2]. The number of motor units was chosen to resemble the first dorsal interosseus (FDI) muscle (100), the abductor digiti minimi (ADM) muscle (300), and the tibialis anterior (TA) muscle (500). The model assumes that the values of P and T do not depend on the actual or preceding ISI [2], [22], [23], [24]. As described in the theoretical section, this does not influence the results in general. The spike trains were generated using a Gaussian distribution for the ISI at different average discharge rates. In the case of correlated activity, the ISI were sampled from a linear combination of common and independent Gaussian noise realizations. The percent of the variance of the common and independent noise provided different levels of correlation between the simulated spike trains. The simulated level of synchronization was independent to the imposed variability for ISI. The simulated torque was obtained by the convolution between the spike trains and the twitch force profile.

### Experimental Protocol

Electrical elicited isometric torques were recorded from the first dorsal interosseus (FDI) in 5 subjects (age: 32±4). The subject was seated on an adjustable chair with the right arm extended in a force brace (Aalborg University). The index finger was fixed in the isometric device for force measurement. The forearm was secured with Velcro straps and the force produced by the index finger was measured with one force transducer (Interface). Electrical stimulation was delivered by a voltage-controlled stimulator (Digitimer, USA). The anode and cathode were placed over the belly of the FDI muscle. The electrical pulses had duration of 1 ms and the average stimulation rate was 8 Hz. Five stimuli with 1 s inter-pulse interval were delivered at the beginning of each trial as a reference. The amplitude of the stimulation pulses (15–36 mA) was selected independently for each subject in order to generate a stable twitch profile. The stimulation intensity were approximately at the maximum, since higher amplitude currents were not generating a significant increase in the amplitude of the twitch responses. The selected stimulation intensity was kept constant during the whole experiment. Pulse trains with different CoVs for ISI (0, 30 and 80%) were used. Electrical stimulation delivered to the surface of the FDI muscle was not selective enough to elicit the activation of a single motor unit twitch. Therefore, we present the experimental results as a compound activity of an undefined number of motor units. This situation is not different, mathematically, to that of a single motor unit twitch train with the compound force as repeating twitches. We used the experiments to validate only the prediction on the effect of twitch fusion.

### Ethics Statements

The experimental protocol was approved by the local ethical committee (Ethikkommission d. Med. Fak. Gottingen, approval number: 1/10/12) in accordance with the Declaration of Helsinki. The subjects provided written informed consent before participation in the experiments.

### Data Analysis

From simulated and experimental spike trains, the twitch profile was estimated using the classic STA technique: the twitch waveform was computed by averaging the torque signal using the simulated or the experimental times of activation as triggers (Eq. 2). In the simulated signals, motor unit short-term synchronization was quantified with the common input strength (CIS) index as the frequency of extra synchronous discharges [27] between all pairs of simulated motor units.

### Availability of Methods and Data

All the simulations reported in this paper can be reproduced using the details reported in the Methods section and the model described in [2]. The experimental data are available upon request to the corresponding author.

## Results

### Theory

**Figure 1:**
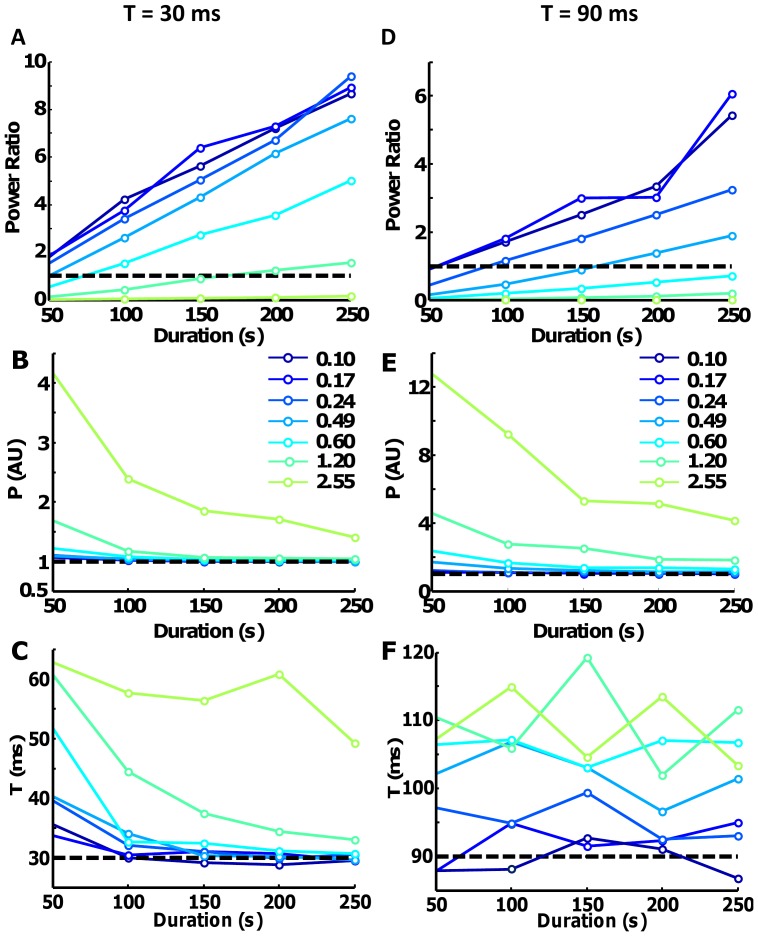
Influence of spike train correlation in the STA estimation. 100 motor unit spike trains were simulated with an average discharge rate of 2∼80%. P was set to 1 and T to 30 (Fig. 1A–C) and 90 ms (Fig. 1D–F) for all units. A–D. The power ratio between the two terms of Eq. 6 is plotted as a function of the duration of the signal used for the calculation and for different levels of synchronization between the simulated spike trains. Each point represents the average of 100 simulations. The synchronization levels (CIS index) were reported at a discharge rate of 8 pps to be comparable with previously reported data in humans. B–E. Similar graphs for estimated values of P and (C–F) T extracted from the STA calculations.

One-hundred motor unit spike trains were simulated (averaged discharge rate (DR) of 2 pps, CoV for ISI ∼80%) and were convoluted with the two twitch waveforms (P = 1 AU; T = 30 ms and T = 90 ms). The low average discharge rate, the CoV for ISI and the time to peak T were chosen to limit the effect of fusion between subsequent twitches (see below). The CIS values were estimated from a similar simulation, but with the average discharge rates of the MU spike trains equal to 8 pps. Due to the influence of the average discharge rates on correlation measures between spike trains [31], this procedure was used to generate CIS values comparable with the values reported in literature that are commonly estimated with similar discharge rates. Moreover, the imposed CoV for ISI was not dependent on the level of synchronization simulated. The results showed that up to a moderate level of synchronization (CIS = 0.10–0.49 pps), the STA estimate is relatively accurate (power ratio >2) with a signal of 100–200 s duration (Figure 1A–D). However, when the synchronization level was relatively high (CIS = 0.60–2.55 pps), the power ratio increased very slowly with the duration of the signal, demonstrating the limitations of the technique even when the observation time (number of triggers) is very large. The estimation of the parameters P and T (Figure 1B–C) were in agreement with the previous simulations, showing almost no distortion in the case of low sincronization (some bias is only present for very short signals, due to the limited number of triggers). In case of T = 90 ms (Figure 1E–F), the results are similar with higher variability in the estimation of the time to peak (T). This is probably due to the lower SNR produced by the larger temporal support of the twitches. However, when the synchronization levels were relatively high, the values of P and T were overestimated in all cases.

**Figure 2:**
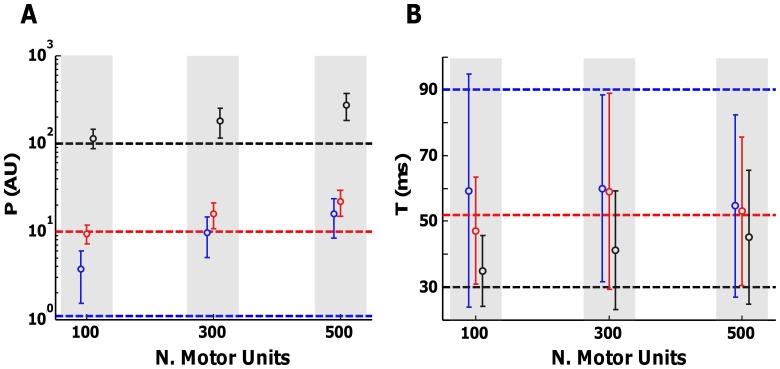
Variability in the STA estimations with different number of motor units active. Simulations were performed with 100, 300 and 500 motor units. The parameters of the twitches were distributed according to an exponential law. Discharge rates and CoV for ISI were fixed at 8% respectively for all motor units. The simulated synchronization level was fixed to have CIS = 0.6 pps. In one case, an intermediate contraction level was simulated in order to have half of the motor units in each pool active. In this scenario, the twitch of the first (blue) and the last (red) recruited motor unit was estimated. In a second case, all motor units were active and the twitch of the last recruited motor unit was estimated. The simulated values are represented with a dashed line. A. Estimations of peak amplitude P in AU. B. Values of time-to-peak T in ms.

The results of a more general simulation are shown in Figure 2, where P and T were simulated according to an exponential distribution [2]. The discharge rates of all motor units were fixed to 8 pps and the CoV for ISI to 15%. A moderate level of synchronization was simulated (CIS = 0.60). The number of motor units was varied between 100 (FDI), 300 (ADM), and 500 (TA). Three scenarios were simulated. First, a recording corresponding to half of the motor units recruited was simulated. In this case, the twitch of the first (blue) and the last recruited motor unit (red) were estimated in 100 simulations. The simulated values are shown with a dashed line. Second, recordings with all the motor units in the pool active were simulated and the twitch parameters corresponding to the last recruited motor unit (black) were estimated using the STA technique. The results showed that P can be well approximated for the last recruited motor unit for the two simulated force levels only in the muscle with 100 motor units (Fig. 2A). A substantial deviation and variability was present for the other two simulated muscles, with a greater number of motor units. For the simulations where the first recruited motor unit was estimated, the deviation was even larger (Fig. 1A), probably due to the fusion effect (see below). In details, for the muscle with 100 MUs the ratio between the simulated and the estimated value of P was 3.6 for the first recruited MU, 0.95 for the last recruited motor unit at the target contraction level and 1.2 for the largest MU. For the muscle with 300 MU the corresponding values were: 9.4, 1.6 and 1.8. For 500 MUs: 15, 2.2 and 2.8. The estimation of the time to peak (T) was poorer than that of P and was accurate only in few cases (Fig. 1B).

**Figure 3:**
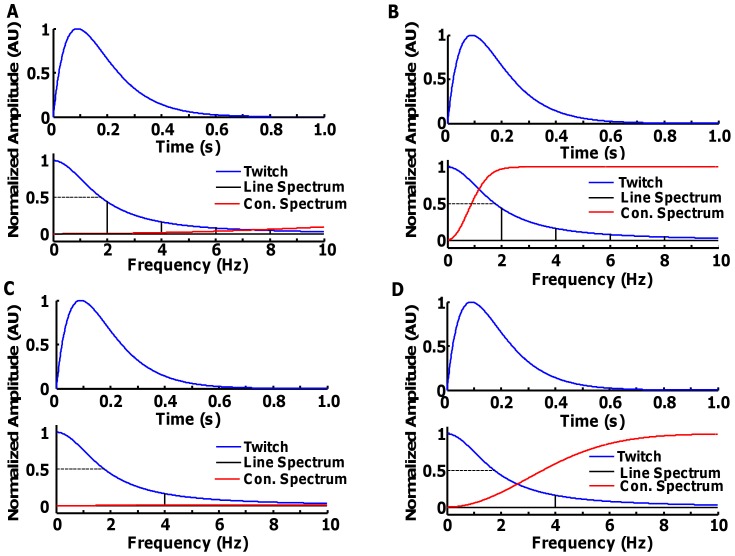
Sampling of the motor unit twitch with different discharge rates and variabilities. The simulated twitch waveform had P = 1 and T = 90 ms (upper plots). A. Period spike train with average discharge rate of 2 pps. B. Same as before but with CoV of approximately 20%. C. Periodic spike train of 4 pps. D. Same as before, with CoV for ISI of 20%.

These results imply that with low levels of synchronization and a relatively long signal, the STA estimate is reliable only with relative low discharge rates and high CoV of ISI. It is interesting to note that the CoV for ISI has a strong influence on the estimate, which is investigated in detail in the following simulations. Eqs. 9–13 describe the limits of the STA technique due to the fusion of the twitches (sampling problem). Figure 3 shows an example of the effect of sampling provided by spike trains with different discharge rates and ISI variability. It shows the power spectra of the motor unit twitch (P = 1, T = 90 ms) compared with the one of the spike train (Eq. 12). For example, even if the frequency of activation is extremely low (2 pps), as in Figure 3A, the spike train provides only few sampling points (line spectra in black) in the bandwidth of the twitch force (the line at 0.5, normalized units, is approximately the −3 dB bandwidth). If a certain amount of ISI variability is introduced, the continuous spectrum (red) previously described improves substantially the sampling because there are an infinite number of frequencies with amplitude different from zero within the bandwidth (Fig. 3C). This additional term has a large frequency bandwidth so that the frequency representation of the train of twitches has components different from DC for any activation frequency. Figures 3B–D show an example of a motor unit with an average discharge rate of ∼4 pps, that is lower than the minimal physiological discharge rate usually observed in humans (6–8 pps). In the periodic case, most of the low frequency components cannot be correctly sampled, since there are no line spectra between DC and 4 Hz. However, with a relatively large ISI variability, the continuous spectrum allows an estimate of the twitch. From this example, theoretically higher discharge rates would require higher CoV for ISI for an accurate estimate of the twitch.

**Figure 4:**
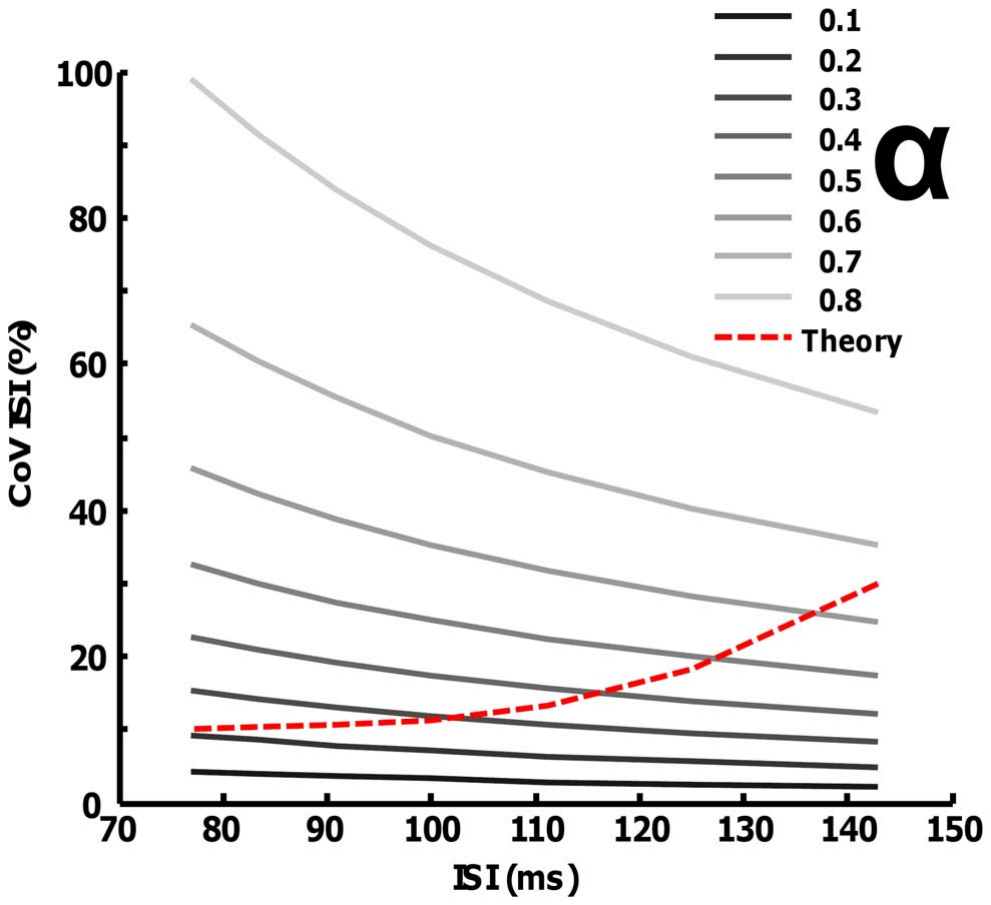
Reconstruction accuracy for different values of ISI and CoV (Eq. A10). The lines for different values of α are plotted as a function of ISI and CoV for ISI (grey lines). An approximation of the relation between ISI and CoV ISI that is usually found in humans was superimposed (red dashed line) for comparison.

In the following and in the Appendix S1 it will be shown that the conditions on the CoV for ISI needed for an accurate twitch estimate imply non-physiological parameters for the discharge pattern. In particular, the continuous spectrum is equivalent to a high-pass filter that sample the transfer function of the twitch. The requirement for a correct sampling is to have the cut-off frequency of this filter near the DC frequency. As reported in the Appendix S1, this is possibile only with relatively high variability of the ISI. Figure 4 shows the estimation of the CoV (Eq. 10 in Appendix S1) that is required to correctly sample 10–80% of the transfer function of a twitch with P = 1 and T = 30 ms (see Appendix S1 for the definition of sampling that has been used). For example, to estimate 50% of transfer function of the twitch, the required CoV varies from 35 to 20% approximately (discharge rates are assumed to be between 7 and 13 pps) (Figure 4). In particular, the estimation of more than the 70% of the twitch waveform requires non-physiological variability values [32] (the red line does not cross the lines corresponding to an alpha of 70 and 80%). Longer twitch contraction times will require even greater variability.

**Figure 5:**
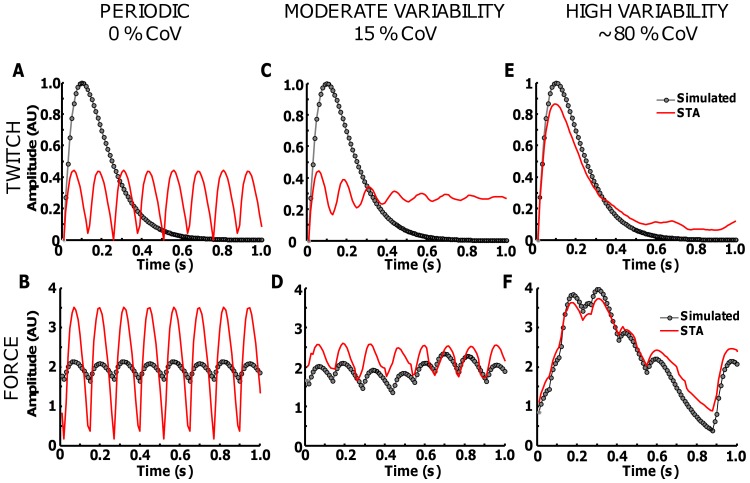
Effect of variability on the twitch fusion when the STA technique is applied. A spike train with DR of 8(0%, 15% and ∼80%) was simulated. The original twitch waveform was simulated with P = 1 and T = 90 ms (grey line in all plots). The STA estimation are shown on the upper plots with red lines. The plots on the bottom show the original force profiles (grey) and the reconstructed force with the estimated STA (red lines).

Figure 5 shows the effect of discharge variability on the estimated twitch waveform and reconstructed force. Spike trains with discharge rate of 8 pps and CoV for ISI of 0 (Fig. 5A–B), 15 (Fig. 5C–D) and 80% (Fig. 5E–F) were simulated. The twitch waveform had P = 1 AU and T = 90 ms. In the periodic case, the STA is completely biased by the effect of the discharge rate (Eq. 12), with a periodic deflection at ∼125 ms (1/DR) (Fig. 5A). In this example, the force cannot be reconstructed by the STA estimation (Fig. 5B). Adding a moderate level of variability, the STA still suffers from a marked error in the estimation (Fig. 5C). Only with very high variability (∼80%), the estimation provided by the STA is accurate (Fig. 5E–F). Nonetheless, such variability cannot be obtained experimentally in voluntary contractions.

**Figure 6:**
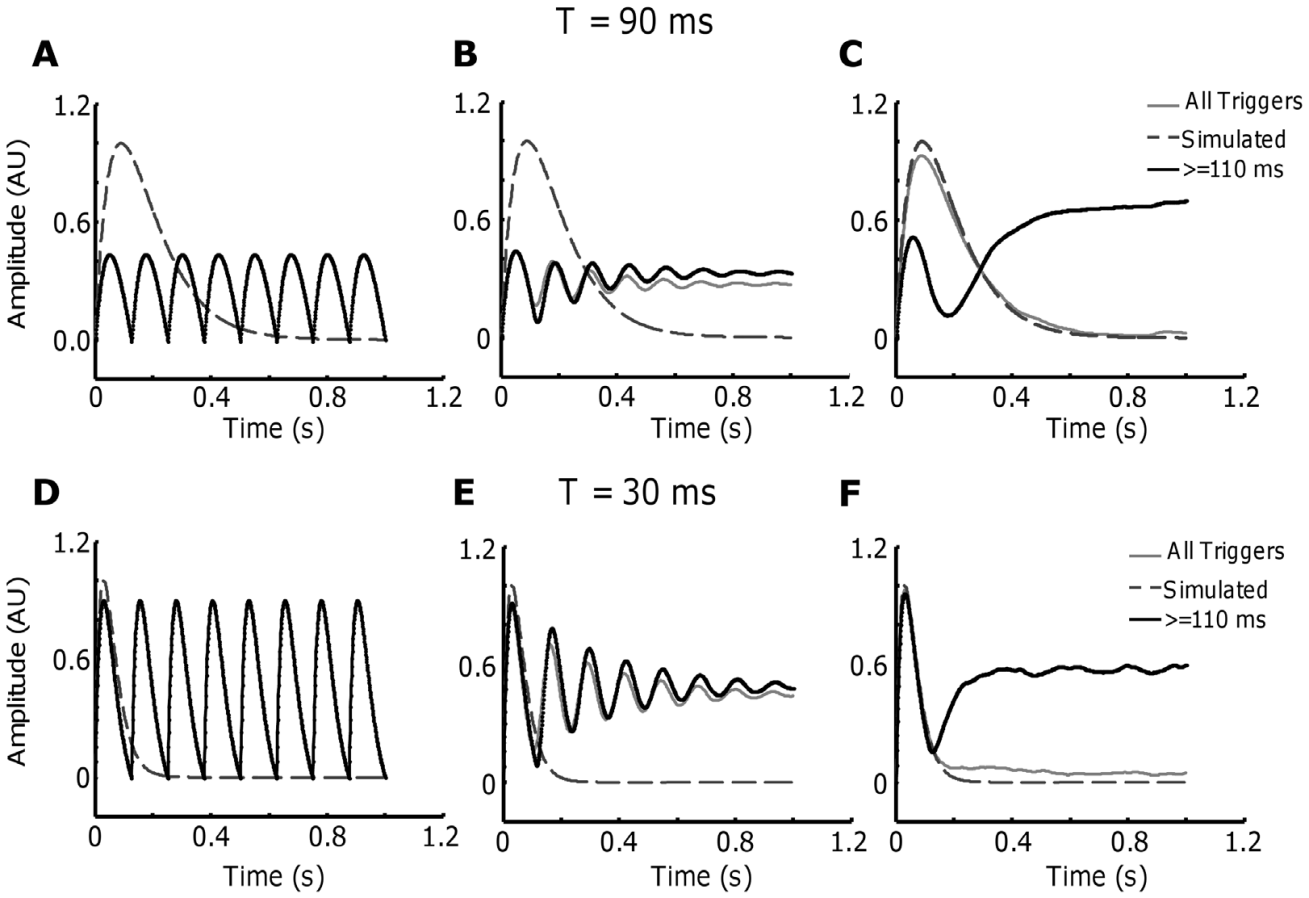
Effect of the selection of the triggers. The twitch waveform has P = 1 and the discharge rate was selected to 8 pps as before. A. Original twitch waveform (blue) with T = 90 ms and estimated twitch with the original (grey) and modified (black) method. B. Similar with CoV for ISI of 15% and ∼80% (C). The estimation for the case with T = 30 ms are reported on the bottom (D, E and F).

A typical approach to mitigate the problem of fusion between twitches is to select only the triggers that are separated by more than a certain interval (e.g., 110 ms [13]) from the preceding and succeeding spikes. Figure 6 shows the effect of this method when twitches are estimated for varying ISI variability. If the twitch train is periodic (8 pps, T = 90 ms), the estimation is poor both with and without selection of triggers (Fig. 6AB), with an average difference from the true twitch of 55%. If a physiological level of variability is introduced, the estimation improves (correlations = 61 vs 56%), but the selection of the triggers does not provide an advantage with respect to using all triggers. For a greater discharge rate (10 pps), the selection of triggers provides advantages with respect to using all triggers, but the improvement is not substantial (correlation: 63 vs 68%). When the level of variability is relatively high (∼80% CoV for ISI), the estimation achieved with all triggers performs considerably better than selecting triggers (99 vs 50%). Therefore, although counterintuitive, trigger selection is in most cases not useful for improving the STA-based twitch estimates.

**Figure 7:**
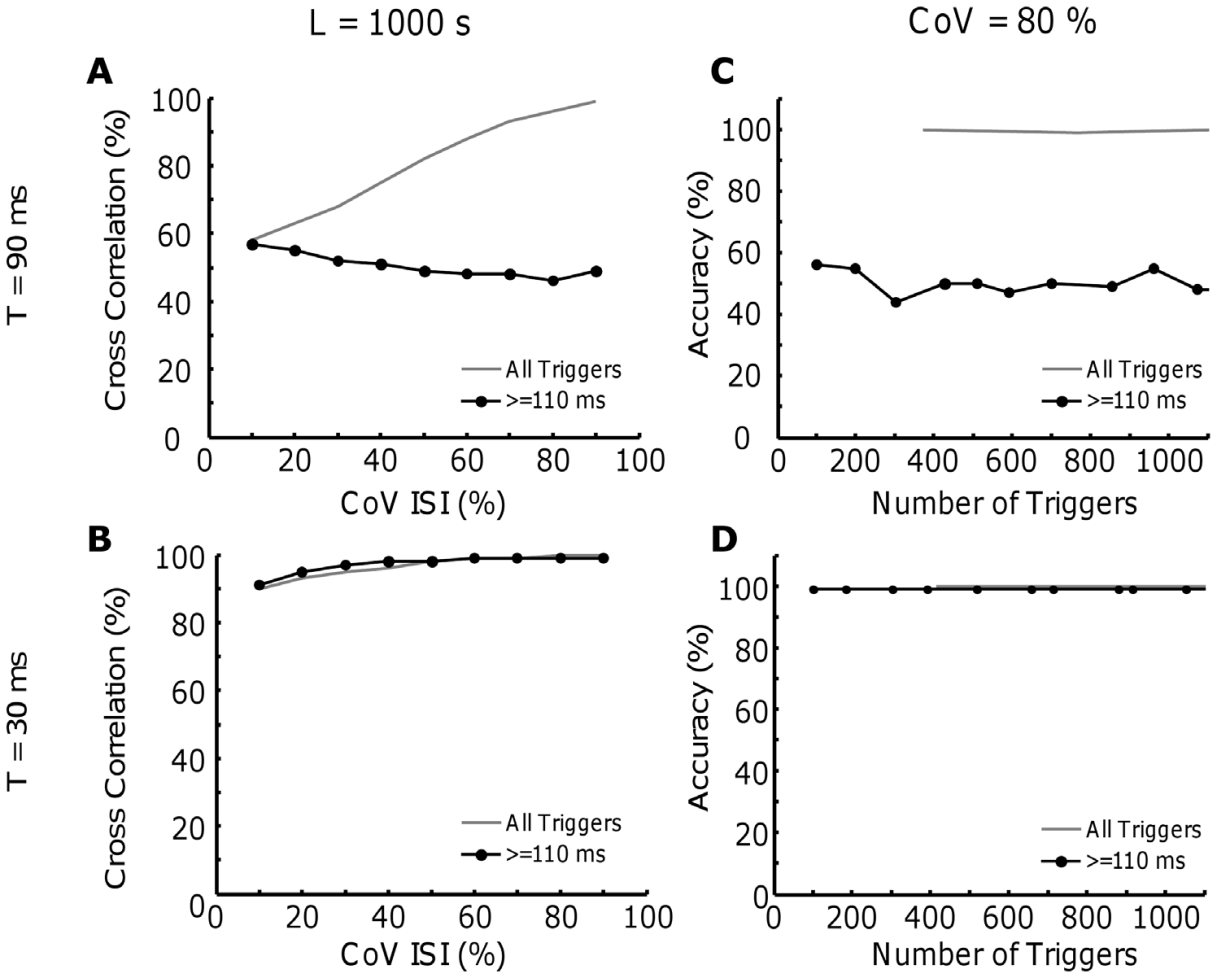
Conseguence of variability and total number of triggers for the estimation of motor unit twitch performed with the original or modified version of the STA technique. A. The cross correlation between the original twitch waveform (P = 1 and T = 90 ms) and the estimation performed with the standard STA technique (black line) or the modified one (pointed line) when the CoV is varied between 10 and ∼80%. B. similar results for T = 30 ms. C. In this case, the CoV for ISI is maintained very high (∼80%), but the number of triggers is increased (T = 90 ms). D. Similar results for the case with T = 30 ms.

Figure 7 shows the general results related to trigger selection. The selection of the triggers was detrimental for the case of T = 90 ms (dotted line), both for the case with a constant number of triggers or a constant CoV for ISI (Fig. 7A–C). In fact, the increase in the variability of the ISI improved the estimation for the without (thick black line) but not with trigger selection. For the case with T = 30 ms the two methods were equivalent for all cases (Figs. 7B–D).

### Experiments

**Figure 8:**
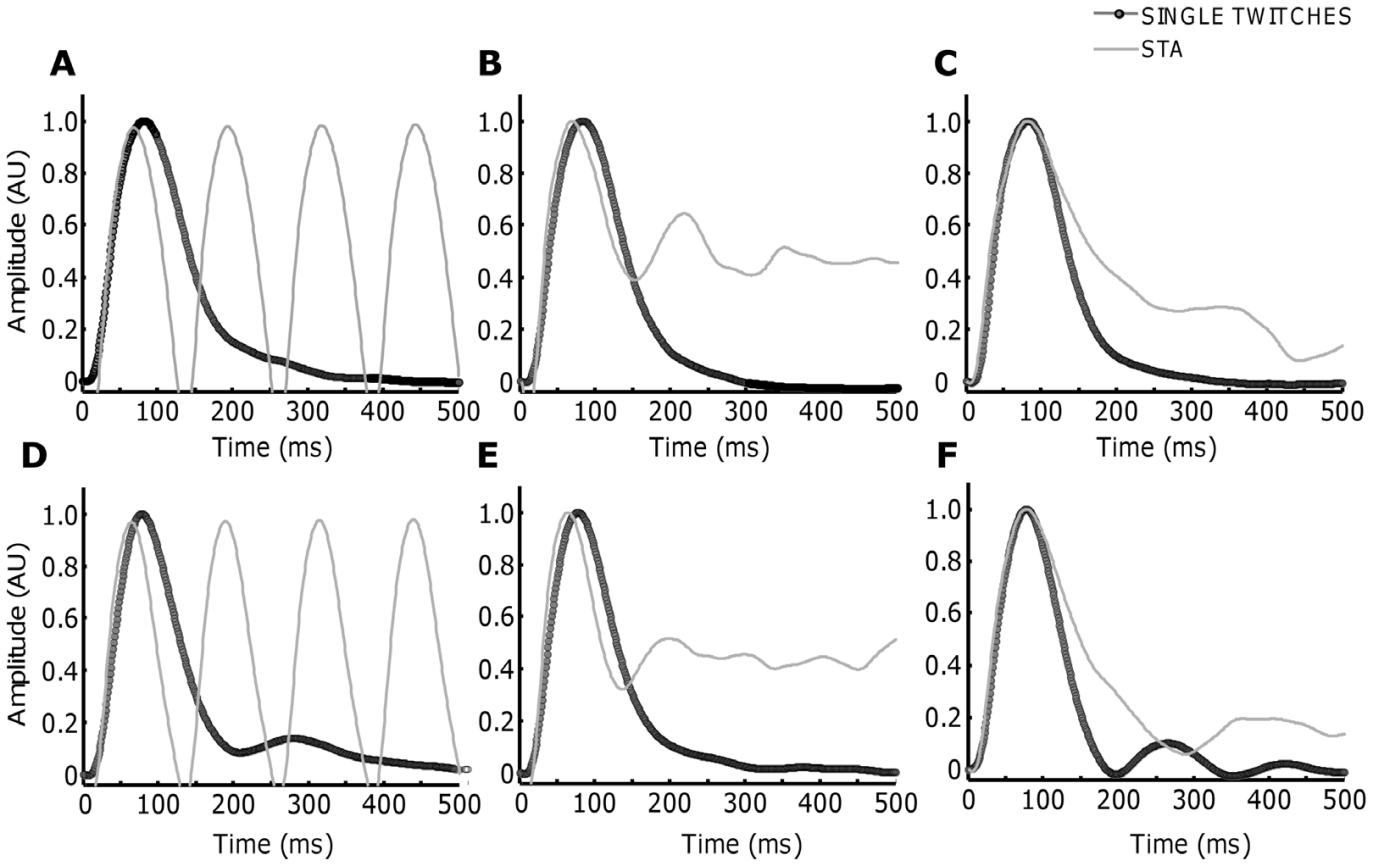
Experimental results performed with stimulation of the FDI muscle subject one (A,B and C) and subject two (D, E and F). Similar as the simulations results, the discharge rate was fixed at 8(black lines). A. Periodic discharge pattern. B. CoV of ∼30%. C. CoV of ∼80%. Similar results are reported for the second subject (D, E and F).

**Figure 9:**
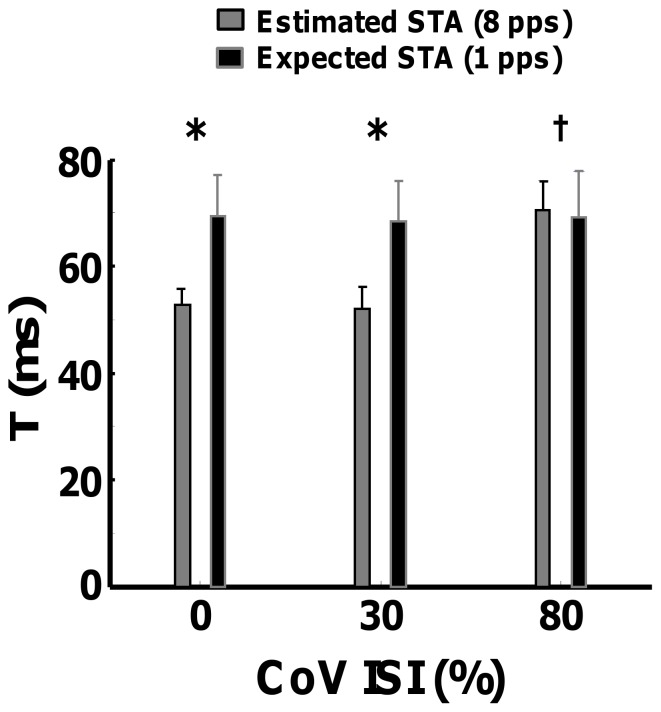
The estimated values of time-to-peak T as a function of the imposed CoV for ISI, averaged across all subjects. Expected (estimation performed as an average of the five pulses generated at the beginning of each trial) and estimated (STA performed in the following 30 s) are shown. T-test analysis was performed for each pair of expected and estimated set of values. *significant at p<0.05.

Electrical stimulation was delivered to the FDI muscles at a constant rate, but with different CoV for ISI. Due to the low selectivity of the electrical stimulation, the compound activity of an indefinite number of motor units was obtained. Figure 8 shows the phenomenon described by the theoretical derivations on the estimated twitch waveforms of electrically stimulated twitches on two representative subjects. Stimulus trains with a rate of 8 pps and CoV for ISI of 0 (Fig. 8A–D), 30 (Fig. 8B–E) % and ∼80% (Fig. 8C–F) were generated. The duration of the stimulation was limited to 30 s to minimize fatigue. In the periodic case, the normalized STA was biased by the effect of the stimulation rate (Eq. 10), with a periodic deflection at ∼125 ms (1/DR). The normalized STA twitch calculated for isolated stimuli (5 stimulations with an interval of 1 s) was considered as the true twitch for comparison with the estimates. Even adding a moderate level of variability, the STA still suffered from a marked error in the estimation (Fig. 8B–E). Only a relatively high variability (CoV ∼80%) allowed accurate estimates (Fig. 8C–F). The minor discrepancies between the quality of the reconstruction for the high variability case (noticeable, longer half-relaxation times), it is probably due to the nonlinear summation of the motor unit twitches. Figure 9 reports the estimated T during the 30-s sustained stimulation at 8 pps and the reference (expected) STA estimation during the 5 pulses at the beginning of each trial for all subjects. The variable T estimated from the reference twitches did not show any statistical difference (P>0.05) between conditions (CoV for ISI equal to 0, 30 and 80%). The same result were found for the peak value. This ensures that the recordings were stable across the entire experimental procedure. The results for the time to peak were in agreement with the theoretical derivations. For low levels of variability, T was significantly underestimated (P<0.05 for CoV of 0 and 30%). However, when a considerable amount of variability was introduced for the ISI, the estimates approached the true values with no significant difference (P>0.05) between the estimated and the expected time to peak.

## Discussion

The estimation of the motor unit twitch forces from the joint force is important to characterize the contractile characteristics of motor units. From the original paper [11], the STA technique has been used for this purpose [6], [33], [34], [35], [36], even if many limitations of the technique have been underlined [12], [13], [14], [37]. In this study, we have shown theoretically the reasons for the limitations that have been previously described empirically by experiments and simulations.

First, we showed that the theoretical expression of the spectrum of the STA (Eq. 6) can describe the influence of the remaining motor unit spike trains (secondary term) on the estimation performed on the triggered motor unit spike train (primary term). In the absence of a significant level of correlation between spike trains, the secondary term reduced to an added uncorrelated noise term that can be mitigated with a suitable number of triggers (Fig. 1), since it increases in power slower than the primary term. However, if the level of correlation is relatively high, the secondary term will increase faster than the primary term, so that the estimate is incorrect for any number of triggers (Fig. 1). Therefore, if a relatively high degree of correlation is present in the muscle, there is a theoretical limit that prevents an accurate estimate of the twitch force by STA, even for infinitively long recrodings. According to our simulations, the level of correlation at which this phenomenon occurs is similar to the one found in the more distal muscles of the upper and lower limbs [38]. However, in specific applications, such as the estimation of the contribution of single motor unit force to multiple directions, the technique may be insensitive to the level of synchronization [20], [39]. In our simulations, the variability in the ISI was not dependent on the level of synchronization imposed. This could be a limitation in our current approach, since the two variables are likely to be related, at least in pathological conditions (e.g. tremor).

Second, we focused on the influence of the spike train characteristics on the fusion of subsequent twitches. Eq. 13 showed that the STA estimation can be seen as a sampling process in the frequency domain, which correspond to a periodization in the time domain. In the case of periodic discharges (Fig. 3A–B), the spectrum of the spike train contains only line spectra that sample the transfer function of the twitch at specific frequencies. More points imply a better reconstruction, therefore lower discharge rates can reconstruct the original twitch better than higher rates (Fig. 3A–C). This was expected since lower discharge rates determine a longer interval between triggers and therefore less fusion between twitches. Less intuitive was the influence of variability in discharge on the estimated twitch. If a certain amount of variability is introduced, the continuous spectrum (Fig. 3B–D) performs a sampling for an infinite number of frequencies. Therefore, depending on the relative amplitude of the continuous spectrum, the STA can provide a more reliable estimation than for pure periodic trains of discharges (Fig. 5A–E). However, in practical situations, the amount of variability that is needed for an accurate estimate with physiological discharge rates is high. For example, Fig. 5E shows that a coefficient of variation of ∼80% would be necessary for an average discharge rate of 8 pps [1], [3], [32]. The experimental results (Fig. 8 and 9) showed a similar behavior as the simulations, demonstrating that the STA technique introduces a considerable amount of error in the estimation when relatively low CoV for ISI are used, but that very high variability may help increasing the estimate accuracy. In physiological conditions, however, the CoV for ISI is only approximately 10–15%. Therefore, although the variability in discharge is useful for obtaining a good estimate of the twitch force, there are no physiological combinations of ISI and CoV for ISI that provide a highly accurate estimate without any twitch fusion. Noneteless, in extreme cases of high threshold motor units with relatively short duration of the twitch and high physiological variability, the performance of the STA may be accettable.

Another interesting observation related to variability is that eliminating from the STA process the triggers that are too close to the preceding and subsequent, as usually suggested [14], is not necessarily advantageous than including all triggers and can be detrimental. Indeed, eliminating triggers decreases the SNR (Fig. 6 and 7) and this decrease is not necessarily compensated by the reduced degree of twitch fusion.

In conclusion, this study provides a theoretical framework for explaining previous experimental results. This framework allows not only interpreting known limitations of the STA, but also understanding behaviors that were not previously described. This includes the influence of variability in spike train and of trigger selection, which have effects different from what previously thought. Moreover, a clear theoretical understanding of the limitations of the STA technique opens the possibility of new approaches to the problem. The theoretical analysis indeed showed that the issue of twitch fusion cannot be solved by a classic avearing approach, not even with trigger selection. Therefore, the only possibility is to apply deconvolution techniques that perform an inversion of the convolutive problem represented in Eq. 1 [21]. These techniques would compensate for the additional convolutional term (Eq. 3) and would eliminate most of the limitations outlined in this study.

## Supporting Information

Appendix S1 (DOCX)
