# Correction: Limitations of the Spike-Triggered Averaging for Estimating Motor Unit Twitch Force: A Theoretical Analysis

**DOI:** 10.1371/journal.pone.0101614

**Published:** 2014-06-27

**Authors:** The PLOS ONE Staff

The second author’s name is spelled incorrectly. The correct name is: Ş. Utku Yavuz. The correct citation is: Negro F, Yavuz ŞU, Farina D (2014) Limitations of the Spike-Triggered Averaging for Estimating Motor Unit Twitch Force: A Theoretical Analysis. PLoS ONE 9(3): e92390. doi:10.1371/journal.pone.0092390
