# Supplementary material for: Limitations of the Spike-Triggered Averaging for Estimating Motor Unit Twitch Force: A Theoretical Analysis
Source: PLoS One. 2014 Mar 25;9(3):e92390. doi: 10.1371/journal.pone.0092390 (PMC3965416; doi:10.1371/journal.pone.0092390)
Supplement: Appendix S1 — (DOCX) [file pone.0092390.s001.docx]

APPENDIX S1 FOR “LIMITATIONS OF THE SPIKE-TRIGGERED AVERAGING FOR ESTIMATING MOTOR UNIT TWITCH FORCE: A THEORETICAL ANALYSIS”

In this Appendix, we derive the analytical expression of the calculation of the variability needed for a reliable estimation of the STA twitch, given a certain value of average discharge rate. If we model the twitch waveform as suggested in [[1](#_ENREF_1)], we have

$g(t)=P\frac{t}{T}e^{1-\frac{t}{T}}$ (S1-1)

where *P* is the peak value and *T* the time-to-peak. If we set *P’ = eP* and *a = 1/T* , we can rewrite S1-1 as

$g(t)=P'ate^{-at}$ (S1-2).

The Fourier transform of Eq. S1-2 is given by [[2](#_ENREF_2)]

$G(f)=\frac{P'a}{(a+j2\pi f)^{2}}$ (S1-3)

with *P’* > 0 and *a* > 0.

Given Eq. S1-3, we want to calculate the frequency value *f_α_* for which the amplitude of the twitch transfer function is equal to *α* % of the maximum amplitude. For this, we solve the following expression:

$\left|\frac{P'a}{(a+j2\pi f)^{2}}\right|-\alpha\left|\frac{P'}{a}\right|=0$ (S1-4).

Eq. S1-4 has the solution:

$f_{\alpha}=\frac{a\sqrt{1-\alpha}}{2\pi\sqrt{\alpha}}$ (S1-5).

Now we focus on the continuous spectrum of Eq. 11:

$Sc(f)=\frac{1}{T_{i}}[1-\left|Q(f)\right|^{2}]$ (S1-6)

where *T_i_* is the period of the discharges of the spike train and *Q(f)* the Fourier transform of the probability density function describing the ISI intervals. If we assume that the ISI intervals are distributed according to a Gaussian function, we have:

$q(t)=\frac{1}{\sigma\sqrt{2\pi}}e^{-\frac{t^{2}}{2\sigma^{2}}}$ (S1-7).

According to [[3](#_ENREF_3)] and [[4](#_ENREF_4)], Eq. S1-7 has a Fourier transform given by

$Q(f)=e^{-\frac{1}{2}(2\pi f)^{2}\sigma^{2}}$ (S1-8).

As before, we can find the value of *f* for which the continuous spectrum is higher than a given value. Since in Eq. 12 the continuous spectrum is multiplied by the transfer function of the twitch, we can calculate the *σ* value so that the amplitude of the continuous spectrum is equal to the amplitude of the twitch transfer function at the frequency *f_α_*. Using a normalized spectrum, we have

$[1-\left|Q(f_{\alpha})\right|^{2}]=\alpha$ (S1-9)

where *Ti* is the average interspike interval and *Q(f)* the Fourier transform of the interspike interval distribution given by S1-8. Now using equation S1-5 and S1-9 we can calculate the value of *σ* so that the continuous spectrum samples at least *α* % of the amplitude of the twitch transfer function:

$\sigma=\frac{\sqrt{-\ln(1-\alpha)}}{2\pi f_{\alpha}}$ (S1-10).

According to Eq. S1-10, for example, with a discharge rate of 7 pps and a twitch with T = 30 ms, the CoV for ISI should be approximately 42 % to sample correctly 75 % of the twitch amplitude.

REFERENCES

1. Fuglevand aJ, Winter Da, Patla aE (1993) Models of recruitment and rate coding organization in motor-unit pools. Journal of neurophysiology 70: 2470-2488.

2. Presti LL, Neri F (1992) L’analisi dei Segnali. Seconda edizione, CLUT, Torino.

3. Abramowitz M, Stegun IA (1972) Handbook of Mathematical Functions with Formulas, Graphs, and Mathematical Tables. National Bureau of Standards Applied Mathematics Series 55. Tenth Printing.

4. Bracewell RN, Bracewell R (1986) The Fourier transform and its applications: McGraw-Hill New York.
